# The Progress of Label-Free Optical Imaging in Alzheimer’s Disease Screening and Diagnosis

**DOI:** 10.3389/fnagi.2021.699024

**Published:** 2021-07-22

**Authors:** Kai Liu, Jiasong Li, Raksha Raghunathan, Hong Zhao, Xuping Li, Stephen T. C. Wong

**Affiliations:** ^1^Translational Biophotonics Laboratory, Systems Medicine and Bioengineering Department, Houston Methodist Cancer Center, Houston, TX, United States; ^2^Department of Gastrointestinal Surgery, The Third Xiangya Hospital of Central South University, Changsha, China; ^3^T. T. and W. F. Chao Center for BRAIN, Houston Methodist Hospital, Houston, TX, United States

**Keywords:** Alzheimer’s disease, label-free optical imaging, spectroscopic imaging, early detection, review

## Abstract

As the major neurodegenerative disease of dementia, Alzheimer’s disease (AD) has caused an enormous social and economic burden on society. Currently, AD has neither clear pathogenesis nor effective treatments. Positron emission tomography (PET) and magnetic resonance imaging (MRI) have been verified as potential tools for diagnosing and monitoring Alzheimer’s disease. However, the high costs, low spatial resolution, and long acquisition time limit their broad clinical utilization. The gold standard of AD diagnosis routinely used in research is imaging AD biomarkers with dyes or other reagents, which are unsuitable for *in vivo* studies owing to their potential toxicity and prolonged and costly process of the U.S. Food and Drug Administration (FDA) approval for human use. Furthermore, these exogenous reagents might bring unwarranted interference to mechanistic studies, causing unreliable results. Several label-free optical imaging techniques, such as infrared spectroscopic imaging (IRSI), Raman spectroscopic imaging (RSI), optical coherence tomography (OCT), autofluorescence imaging (AFI), optical harmonic generation imaging (OHGI), etc., have been developed to circumvent this issue and made it possible to offer an accurate and detailed analysis of AD biomarkers. In this review, we present the emerging label-free optical imaging techniques and their applications in AD, along with their potential and challenges in AD diagnosis.

## Introduction

Alzheimer’s disease (AD), the most common form of dementia, accounts for 60 to 80% of dementia cases (No authors listed, 2021). AD is characterized by progressive impairments of cognition and memory due to irreversible damage of brain neuronal cells. It was the sixth leading cause of death in the United States before 2020, causing nearly 120,000 annual deaths and imposing substantial emotional and financial burdens on families and society (Ahmad and Anderson, 2021). In 2020, the death toll rose to 130,000 (Ahmad and Anderson, 2021). An estimated 6.2 million Americans age 65 and older are living with Alzheimer’s dementia and will cost $355 billion to the United States in 2021. The number of people aged 65 and older with Alzheimer’s dementia is projected to grow to 13.8 million by 2060, and the cost of caring for them to $1.1 trillion by 2050 (No authors listed, 2021).

Although the two classical hallmarks, extracellular senile plaques (SPs) aggregated by amyloid beta-peptide (Aβ) around the neurons and intraneuronal neurofibrillary tangles (NFTs) embodying hyperphosphorylated tau protein (p-tau), were confirmed for AD decades ago (Kosik et al., 1986; Yamada et al., 1988), the neuropathogenesis of AD has not been fully elucidated. Clinical symptoms of AD include memory loss, cognitive decline, behavioral disorder, changed personality, and visual disorders. According to the severity degree of manifestation, AD can be divided into five phases: preclinical AD, mild cognitive impairment (MCI), mild AD, moderate AD, and severe AD. Currently, there is no effective therapy to stop or reverse the disease progression. AD drugs approved by the U.S. Food and Drug Administration (FDA), such as acetylcholine esterase inhibitors, only aim to improve mild-moderate AD patients’ symptoms (No authors listed, 2021). Extensive clinical trials have been conducted on AD therapeutics based on eliminating classical neuropathologies, such as Aβ and its aggregation (Vandenberghe et al., 2016; Ostrowitzki et al., 2017; Relkin et al., 2017). Most of them recruited mild to moderate AD patients and did not show any efficacy (Pais et al., 2020). It is postulated that neural dysfunction and death happen long before any apparent symptoms, and thus the removal of Aβ plaques cannot reverse the damage that has already occurred. Aducanumab was proven to slow down the AD process in the clinical trial and under FDA’s review, but only for MCI and early AD patients (Schneider, 2020; No authors listed, 2021). Other early AD trials are still ongoing (Pais et al., 2020). Therefore, screening for early AD may be the pivotal precondition for the efficacy of AD drugs. Furthermore, an ideal screening tool would help AD clinical trials with more rapid and larger sample-sized patient recruitment and speed up AD drug development. Besides, an early diagnosis can help AD patients get more benefits from available therapies and sustain a good quality of life for longer (Rasmussen and Langerman, 2019).

Clinical diagnosis of AD mainly consists of three parts: neuropsychological tests, cerebrospinal fluid (CSF) test, and neuroimaging (McKhann et al., 2011; Jack et al., 2018). The neuropsychological tests, including Mini-Mental State Examination (MMSE) and the Alzheimer’s Disease Assessment Scale-Cognitive Subscale (ADAS-Cog), evaluate disease progression and treatment response. However, it is difficult for them to separate AD from other types of dementia, especially for incipient AD (Storey et al., 2002). Aβ_1__–__42_, p-tau, and total-tau protein (t-tau) have been used as the standard biomarkers in the CSF test (Jack et al., 2018), while other biomarkers in CSF and examinations of other biofluids, including blood, oral fluids, ocular fluids, and olfactory fluids, are under active investigation (Lee et al., 2019). The invasiveness of the CSF testing limits its applicability, however. Combining multiple biomarkers in biofluid tests can achieve higher specificity but would cost more. Several neuroimaging techniques have been developed for AD diagnosis, including structural MRI (sMRI), functional MRI (fMRI), [18F] fluorodeoxyglucose PET (FDG-PET), Aβ-PET, and tau-PET. SMRI and fMRI are non-invasive tools for the diagnosis of AD and assessment of the disease progression through the evaluation of structural abnormality (atrophy) and brain activity (cerebral blood flow) (Dona et al., 2016). FDG-PET can also evaluate brain activity by monitoring glucose metabolism (Lotan et al., 2020). Nevertheless, these imaging modalities are not ready to certainly differentiate AD from other types of dementia due to the deficiency of AD-specific biomarkers. In contrast, amyloid-PET and tau-PET achieve a differential diagnosis of AD by detecting aggregated Aβ and tau neurofibrillary tangles with radiotracers (Matsuda et al., 2019). However, the widespread implementation of these PET diagnostic imaging techniques is limited due to their high costs, low contrast, long acquisition time, low spatial resolution (around 2.5 mm), and in rare instances, causing major allergic reactions due to the radioactive trackers ingested or injected (Cho et al., 2008). Non-invasive, high resolution, high contrast, and cost-effective methods are lacking in the early screening and diagnosis of AD.

The aforementioned diagnosis methods are just auxiliary proofs to increase the certainty of possible or probable AD dementia. Currently, the characteristic AD neuropathology is the only gold standard of AD diagnosis. Multiple exogenous reagents have been developed for visualizing biomarkers and their subtle changes at the optical microscopy level, including fluorescent dyes (e.g., Congo red, Thioflavin S, etc.) and antibodies that are used for the immunohistochemical detection of biomarkers, especially in *ex vivo* and *in vitro* studies of AD (Inouye and Kirschner, 2005; Wei et al., 2005). Their appearance enables high spatial resolution and high imaging contrast, but several drawbacks limit their usage. Before observation, the sample preparation includes fixation, embedding, dissection, and staining, which is time-consuming and labor-intensive. The preparation process also damages the underlying biological structure of samples to the extent that observed results might not be meaningful. Furthermore, it has been reported that these exogenous reagents may interfere with the formation of biomarkers (Lee, 2002; Cohen et al., 2009), often leading to inaccuracy or even contradicting results. Label-free optical imaging can circumvent these issues.

**FIGURE 1:**
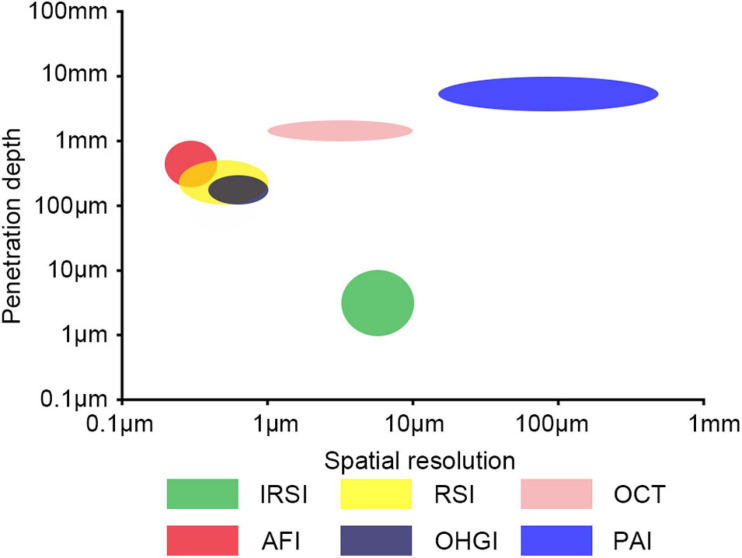
Label-free optical imaging modalities as function of penetration depth and spatial resolution. IRSI, infrared spectroscopic imaging; RSI, Raman spectroscopic imaging; OCT, optical coherence tomography; AFI, autofluorescence imaging; OHGI, optical harmonic generation imaging; PAI, photoacoustic imaging.

Several label-free optical imaging techniques with high spatial resolution have been developed, including infrared spectroscopic imaging (IRSI), Raman spectroscopic imaging (RSI), Optical Coherence Tomography (OCT), autofluorescence imaging (AFI), and optical harmonic generation imaging (OHGI) (Figure 1; Kodach et al., 2010; Campagnola, 2011; Andrew Chan and Kazarian, 2016; Krafft et al., 2016; De Bruyne et al., 2018; Leitgeb and Baumann, 2018; Yeh et al., 2018; Beć et al., 2020). Label-free optical techniques could give reliable and precise results with high resolution, high intrinsic contrast, and less sample preparation, and thus have the potential to be effective and non-invasive methods for early screening and diagnosis of AD. In this paper, we review the present applications of label-free optical imaging in AD and discuss their potential and challenges for AD diagnosis.

## Infrared Spectroscopic Imaging (IRSI)

Infrared spectroscopic imaging (IRSI) based on infrared spectroscopy (IR), also called IR chemical imaging, is sensitive to the structure of molecules. IRSI has been broadly applied in medicine, including various cancers and neurodegenerative diseases (Pilling and Gardner, 2016). This section is divided into two parts: the first part is the principle and applications of IR spectroscopy in AD, which forms the foundation of infrared spectroscopic imaging, and the second part is the application of IRSI in AD.

### Infrared Spectroscopy in AD

Infrared spectroscopy is based on the electric dipole moment of molecular bonds. Molecules have specific absorb frequencies, also called resonant frequencies, which means absorption happens when the molecules are irradiated by a specific frequency of IR light. Thus, the absorbed IR wavelength and the corresponding absorbance can be recorded to analyze the type and contents of molecules (De Bruyne et al., 2018). IR spectroscopy can be classified according to spectral regions as near-IR spectroscopy (13,000–4000 cm^–1^), mid-IR spectroscopy (4000–400 cm^–1^), and far-IR spectroscopy (<400 cm^–1^). Mid-IR spectroscopy is essential for biological specimen analysis. Critical and characteristic regions in the mid-IR spectrum include the fingerprint region (1500–600 cm^–1^), which is unique for each given molecule, and the amide I and amide II (amide I/II) region (1500–1700 cm^–1^), which is used to recognize the secondary structure elements of molecules, e.g., random coils, α-helices, and β-sheets (López-Lorente and Mizaikoff, 2016; De Bruyne et al., 2018). IR spectroscopy has been frequently deployed in AD research based on the specific spectra of AD-relevant biomarkers such as Aβ, p-tau, and their fibrils. The absorbance range in the amide I region for different secondary structure elements for AD-relevant biomarkers is summarized in Table 1. Different processing methods for the raw infrared data determine the type of IR spectroscopy. The most common IR spectroscopy in AD research is Fourier-transform infrared spectroscopy (FTIR), which can be categorized into transmission Fourier-transform infrared spectroscopy (transmission FTIR), reflection absorption IR spectroscopy (RAIRS), and attenuated total reflection Fourier-transform infrared spectroscopy (ATR-FTIR), depending on the sampling modes.

**TABLE 1:** The assignments of amide I band positions in FTIR spectra to secondary structures.

Secondary Structure	Band Position in H_2_O/cm^–1^	Band Position in D_2_O/cm^–1^
α-helix	1648–1657	1648–1658
Disordered	1642–1655	1640–1650, 1663–1668
β-sheet	1620–1639	1617–1640
Anti-parallel β-sheet	1676–1695	1680–1696
β-turn	1660–1695	1665–1696

#### Transmission Fourier-Transform Infrared Spectroscopy

Transmission FTIR spectroscopy (also called conventional FTIR spectroscopy) is among the earliest spectroscopy techniques applied to Alzheimer’s research. Since the early 1990s, several transmission FTIR studies have been conducted on the conformation of Aβ relevant fibrils *in vitro* (Fraser et al., 1991; Howlett et al., 1995; Soto et al., 1995; Guo and Wang, 2012). The dominant role of β-sheet structures in fibrils has been repeatedly verified. Transmission FTIR was employed to analyze the conformation of aggregates of various synthetic Aβ isoforms and Aβ fragments for investigating the mechanism of Aβ deposition. For instance, the aggregates of Aβ fragments including Aβ_1__–__25_, Aβ_25__–__35_, and Aβ_33__–__42_ were analyzed by transmission FTIR (Guo and Wang, 2012). The results showed that the Aβ_1__–__25_ favored the formation of the fibrils with the anti-parallel β-sheet structures, Aβ_25__–__35_ was prone to form fibrils with parallel β-sheet structures, and Aβ_33__–__42_ formed the fibrils with both anti-parallel and parallel β-sheet structures. These results suggested that charged residues affect the conformation of aggregates of Aβ fragments. Benseny-Cases et al. (2007) monitored the process of the aggregation of the synthetic Aβ_1__–__40_ with transmission FTIR. In this study, the spectrum of Aβ oligomers in the aggregation process showed two peaks at 1626 and 1645 cm^–1^, representing β structures and a mixture of unordered and helical structures, respectively. The results suggested that the Aβ oligomers contained non-fibrillar β structures, which would be transformed into fibrillar β structures. Transmission FTIR was further used to analyze Aβ fibrils’ conformation to investigate the influence of microenvironment factors for Aβ aggregation, such as pH and metal ions (Fraser et al., 1991; Sivakumar et al., 2012).

**FIGURE 2:**
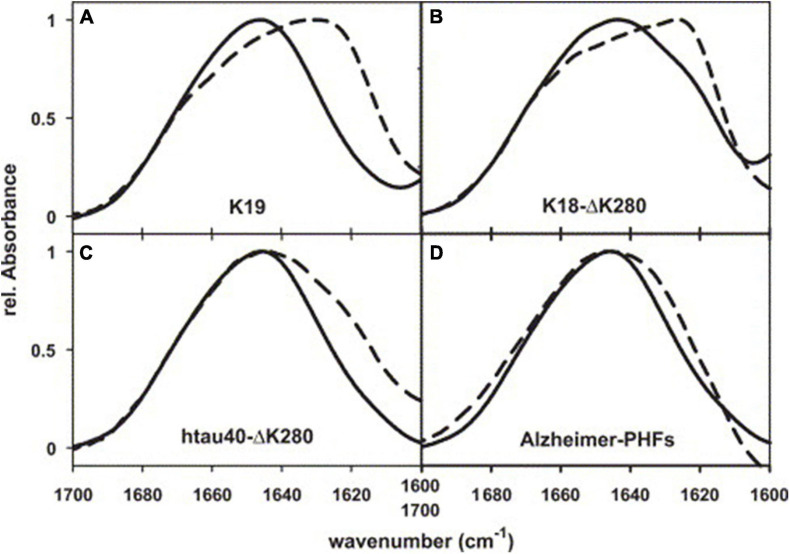
FTIR spectra of PHFs from Alzheimer’s brain tissue, reassembled from recombinant tau, and soluble tau. **(A)** Three-repeat construct K19, **(B)** four-repeat construct K18-ΔK280, **(C)** full-length isoform htau40-ΔK280, **(D)** AD-PHFs. Dashed curves, PHFs; solid curves, soluble tau. Reprinted with permission from von Bergen et al. (2005). Copyright (2004) Elsevier B.V.

The initial transmission FTIR study on paired helical filaments (PHFs), which are compositions of neurofibrillary tangles (NFTs) and aggregates of tau proteins, showed no β-sheet in PHFs with a peak observed at 1658 cm^–1^ in the spectrum (Schweers et al., 1994). This was confirmed in another study in which the spectrum of PHFs showed a peak at 1654 cm^–1^, which was assigned to α helices (Sadqi et al., 2002). However, this conclusion was questioned by von Bergen et al. (2005) who investigated the shifts in the formation of PHFs by analyzing four kinds of PHFs with transmission FTIR (Figure 2). Each shows a peak or a shoulder at about 1630 cm^–1^ compared with the spectrum of soluble tau protein, which means an increase of β-sheet structure in the aggregation of PHFs contrary to previous studies (Schweers et al., 1994; Sadqi et al., 2002). In another study, Zhu et al. (2011) incubated human tau_244__–__372_ fragments in the presence and absence of Pb^2+^ and analyzed the fibrils with FTIR. The results manifested an increase of the band at 1630 cm^–1^ for fibrils (presence of Pb^2+^) compared to the absence of Pb^2+^, indicating that Tau_244__–__372_ fibrils (presence of Pb^2+^) contained more β-sheet structures, while also suggesting that Pb^2+^ promoted the aggregation of Tau_244__–__372_. The results may imply a connection between heavy metal pollution and AD.

Although transmission FTIR has shown usefulness in AD research, there are several limitations. First, the sample must be transparent as infrared light restricts the sample type and the opacity problem will affect the spectrum. Second, the sample preparation is complex and time-consuming, affecting its reproducibility. Last, the two IR transparent windows that are necessary for sample deposition are fragile and expensive. RAIRS and ATR-FTIR were developed for these issues.

#### Reflection Absorption Infrared Spectroscopy

Reflection absorption infrared spectroscopy (RAIRS), also called infrared reflection absorption spectroscopy (IRRAS) or transflection mode IR spectroscopy, is a basic reflectance FTIR in which the infrared irradiation is transmitted through the sample, reflects at the surface of the substrates, and crosses the sample again to the detector. Considering that infrared light in RAIRS goes through the sample twice, RAIRS is more sensitive than transmission FTIR. Furthermore, sample processing for RAIRS is simpler than transmission FTIR, without any window preparation. It is a classic instrument to investigate the adsorption of molecules on solid/liquid interfaces, especially for the interaction between protein and lipid monolayers. Therefore, most of its applications in AD study the adsorption interaction of Aβ with the surface of membranes and other biomaterials *in vitro*, targeting the mechanism of Aβ aggregation and novel adsorption-based detection and therapy (McMasters et al., 2005; Ragaliauskas et al., 2014; Matsubara et al., 2018). Matsubara et al. (2018) prepared two lipid membranes (monosialotetrahexosylganglioside (GM1)-enriched membranes and glucosylceramide (GlcCer)-enriched membranes) for inducing the aggregation of Aβs and employed RAIRS to analyze the conformation of induced Aβ fibrils. The spectra of these Aβ fibrils suggested that the GM1-enriched membranes induced parallel β-sheets whilst GlcCer-enriched membranes induced the anti-parallel β-sheets. In another study, the RAIRS spectrum revealed that the adsorption of Aβ_1__–__42_ oligomers onto octadecanethiol (ODT) membranes induced a transformation from β-sheet rich structures to β-sheet depleted structures, which suggested the therapeutic potential of CH3 terminated membranes through adsorbing Aβ oligomers and reversing the aggregation process (Ragaliauskas et al., 2014). However, an intrinsic issue for RAIRS is an electric field standing wave, which may lead to a deviation of spectral interpretation (Bassan et al., 2013).

#### Attenuated Total Reflection Fourier-Transform Infrared Spectroscopy

As a special reflectance FTIR, ATR-FTIR takes advantage of accessories called internal reflection element (IRE) crystals with high refractive indices (e.g., diamond, zinc selenide, and germanium), which also contribute to the high spatial resolution of ATR-FTIR. The evanescent waves produced when the IR irradiation is reflected at the inside surface of the crystals penetrate the samples, which have direct contact with IRE crystals. The unabsorbed evanescent waves are detected as the basic data for the generation of the spectrum. The penetration depth of evanescent waves is between 1 and 2 μm, limiting its sample thickness and detection range. The sample thickness should be greater than 2 μm to avoid spectral artifacts, and only the surface of samples can be analyzed. However, the ATR-FTIR has been popular due to its high sensitivity and simple sample preparation, which only requires direct contact between samples and IRE crystals. Therefore, ATR-FTIR was frequently used in multiple AD research areas in recent years, including the structure of AD relevant proteins and fibrils (Mendieta et al., 2005; Cerf et al., 2009; Bisceglia et al., 2018), AD diagnosis based on biological fluid (Nabers et al., 2016; Depciuch et al., 2019), and AD treatment research (Schartner et al., 2017).

A specific characteristic of ATR-FTIR is its ability to determine the orientation of the second structure of membrane proteins (Goormaghtigh et al., 1999), which benefits AD research in which the synthesis and aggregation of Aβ have been connected with membranes (Butterfield and Lashuel, 2010). Two studies utilized ATR-FTIR and revealed that the ganglioside clusters in the membrane promoted the aggregation of Aβ_1__–__40_ with an anti-parallel β-sheet structure, which is a kind of toxic amyloid fibril (Matsuzaki and Horikiri, 1999; Fukunaga et al., 2012). With ATR-FTIR, Koppaka and Axelsen (2000) found that the oxidatively damaged phospholipid membranes prompted the aggregation of Aβ_1__–__42_, and the orientation of the β-sheet plane was parallel to the membrane. Based on this study, another study performed by the same team revealed that the combination of Aβ_1__–__42_ and oxidatively damaged phospholipid membranes could promote the aggregation of Aβ_1__–__40_ (Koppaka et al., 2003). Kakio et al. (2004) compared the structure of Aβ aggregates formed in solution with membranes. There were considerable differences between the spectra of the Aβ aggregates formed in solution and membranes. The results suggested a significant structural distinction that Aβs form anti-parallel β-sheets in membranes and parallel β-sheets in solution. As an integral membrane protein that generates the Aβ, amyloid precursor protein (APP) is the perfect subject for investigation by ATR-FTIR. In a membrane model, ATR-FTIR spectra indicated the transmembrane domain of APP is a helical structure with an angle of approximately 20° (Itkin et al., 2017). Therefore, ATR-FTIR has been the mainstream mode of FTIR in AD research as a result of its superiority of simple sample processing and low limitation of sample thickness.

### Infrared Spectroscopic Imaging in AD

Infrared spectroscopic imaging is the combination of Fourier transform infrared spectroscopy and light microscopy, also called Fourier transform infrared microscopy (FTIRM). It is a special FTIR to recognize and spatially resolve the chemical composition of tissues *in situ*. Compared to a thermal light source (e.g., globar light) for conventional FTIRM, the synchrotron source with much higher brightness allows a much better spatial resolution, supporting FTIRM to observe tissue slices at a subcellular level. Therefore, synchrotron FTIRM is suitable for the research of AD histopathology. Choo et al. (1996) first visualized the Aβ deposits in human brain tissue with synchrotron FTIRM. The center of Aβ deposits was highlighted with high spectral intensity at 1632–1634 cm^–1^, corresponding to β-sheet conformation. In this study, the author compared the synchrotron FTIRM with conventional FTIRM. Based on the guarantee of high-quality spectra, the conventional FTIRM only achieved a limited spatial resolution between 24 μm × 24 μm and 50 μm × 50 μm, while the synchrotron FTIRM could provide a higher spatial resolution of 12 μm × 12 μm.

**FIGURE 3:**
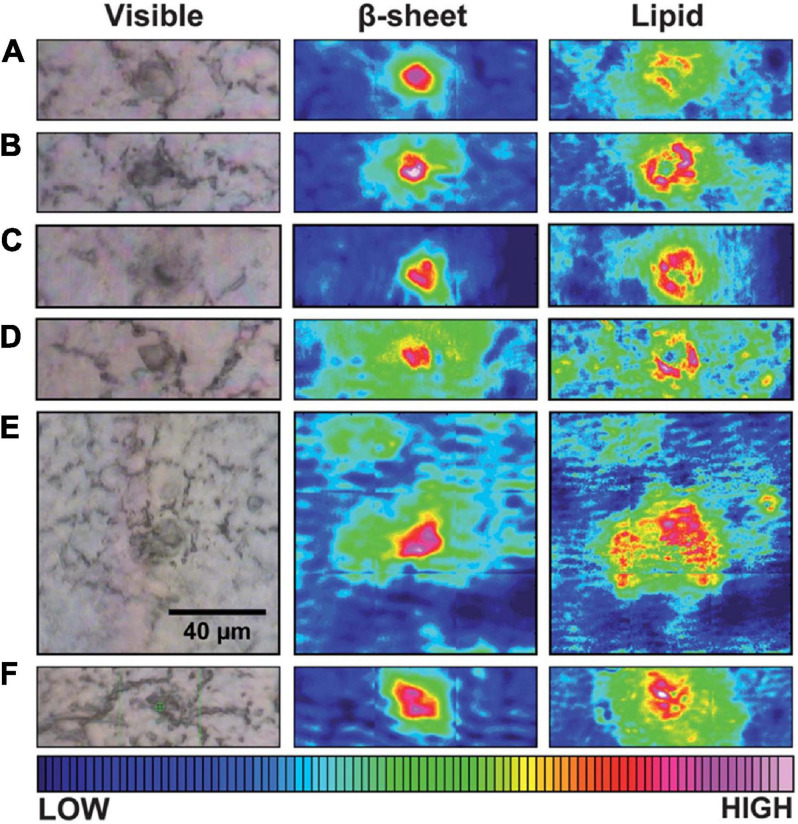
Typical visible and FTIR images of unstained tissue containing dense core plaque observed in three different CRND8 mice aged 5 months **(A–D)** and two aged 8 months **(E,F)**. Left column: dense core appears darker than surrounding tissue under white light. Central column: aggregated plaque is shown from the intensity of β-sheet shoulder. Right column: plaques are surrounded by a lipid membrane-like signature that infiltrates to the core of all plaques, at this spatial resolution. Scale bar = 40 μm and applies to all images. Color bar indicates low (blue) to high (red) for component imaged. Reprinted with permission from Liao et al. (2013). Copyright (2013) The Royal Society of Chemistry.

In contrast with the amide I peak (around 1630 cm^–1^) for imaging of Aβ plaques, the CH_2_ stretch peak (around 2900 cm^–1^) helps visualize lipids in IR imaging. In one study, brain tissue sections from two transgenic AD mouse models (*TgCRND8* and *3 × Tg*) and an AD patient were successively analyzed by synchrotron FTIRM (Liao et al., 2013). Their IR maps suggested that the dense core plaques are surrounded and infiltrated with phospholipids which are membrane components (Figure 3). Furthermore, according to the FTIR map of the 1740 cm^–1^/2960 cm^–1^ ratio (lipid oxidation) and 1630 cm^–1^/1650 cm^–1^ ratio (peptide aggregation), the lipids surrounding plaques have a higher level of oxidation in AD patients than in non-AD controls, which may indicate a critical role in AD process (Benseny-Cases et al., 2014). In addition to lipids, the colocalization of metal ions and Aβ plaques was achieved through the combination imaging of synchrotron FTIRM and synchrotron X-ray fluorescence (SXRF) microscopy (Miller et al., 2006; Leskovjan et al., 2009). The accumulation of Zn and Cu was co-localized with the Aβ plaques in human AD brain tissues, suggesting a relationship between metal ions and Aβ plaque formation in AD (Miller et al., 2006). The same experiment on AD transgenic mice (*PSAPP* mice) showed different results, with a 29% increase of Zn and a decrease of other metal ions (including Cu, Fe, and Ca) in Aβ plaques, revealing a difference between the AD mouse model and AD patients (Leskovjan et al., 2009).

The distribution of creatine in brain tissues of AD transgenic mouse models was also visualized through synchrotron FTIRM with the intensity of bands around 1300 cm^–1^ (Gallant et al., 2006; Kuzyk et al., 2010). Compared with the control brain, much more creatine deposits were found in the brain samples of both AD mice models and patients, suggesting that the elevation of creatine may play a role in AD progression and may serve as a biomarker for AD diagnosis. Due to its function of exploring the chemical change of the brain tissue of AD models or patients, synchrotron FTIRM has been a valuable technique to test potential treatments in AD mouse models, such as *Lepidium sativum* (Ahmed et al., 2020).

## Raman Spectroscopic Imaging (RSI)

The basis of Raman spectroscopic imaging (RSI) is Raman spectroscopy, which is vibrational spectroscopy for detecting biomarkers by monitoring molecular vibrations. Specifically, Raman spectroscopy is based on the inelastic scattering of light discovered by C. V. Raman (Raman and Krishnan, 1928). This phenomenon is called Raman scattering. After scanning a sample with monochromatic light, the detected scattered radiation differs from the incident light in frequency and power because of the interaction between the incident light and the chemical bonds of a molecule in the sample. The frequency difference is called the Raman shift, which depends on the compositions and chemical bonds. The Raman spectrum can ideally help to analyze the compositions and molecular structures of samples, both *in vitro* and *ex vivo*. With the assist of coupled microscopes and suitable data processing, Raman imaging platforms are constructed for the high-resolution visualization of histopathology. In this section, both the experimental application of Raman spectroscopy and the present use of Raman spectroscopic imaging in AD are presented. In the former part, multiple Raman spectroscopy techniques are briefly introduced as a foundation for the latter part, in which the feasibility of Raman spectroscopic imaging on AD tissues is provided.

### Raman Spectroscopy in AD Research

Raman spectroscopy’s ability to distinguish AD tissues from normal tissues relies on recognizing AD-specific molecular shifts, including the accumulation of Aβ fibrils, tau fibrils, and other metabolic changes. Raman data can be readily interpreted in AD research fields (Table 2). Comparing with the interference of H_2_O for IR spectroscopy, Raman spectroscopy averts this problem with weak Raman scattering of H_2_O, which means less sample preparation for Raman spectroscopy than IR spectroscopy (Kong and Yu, 2007; Lopes et al., 2016).

**TABLE 2:** The assignments of band positions in Raman spectra (Dong et al., 2003; Sudworth and Krasner, 2004; Chen et al., 2009; Ramachandran et al., 2014; Xiong et al., 2014; Xiong and JiJi, 2017).

Assignments	Band positions/cm^–1^	Assignments	Band positions/cm^–1^
Phenylalanine	621, 1001–1004, 1031, 1171, 1208–1211, 1585, 1604–1608	Amide III, β-sheet	1220–1245
Tyrosine	642, 829–830, 850, 1171–1177, 1208–1211, 1608	Amide III, disordered	1240–1279
Methionine sulfoxide	704–710, 1010	Amide III, α-helix	1267–1270, 1293–1300
Alanine	763	C-H deformation (lipid and protein)	1298–1300, 1440–1449
Skeletal C–C stretch (cholesterol)	1064–1065	Amide S, disordered	1380–1393
Arginine, Lysine	1080	Amide S, β-sheet	1395–1406
Asparagine, Glutamine	1080, 1402	CH_2_ bending (protein)	1455–1466
C-N stretch	1080, 1124–1130, 1153	Amide II, disordered	1548–1561
C-C stretch	1080–1088, 1129–1130	Amide II, β-sheet	1550–1564
PO^2–^ symmetrical stretch	1088	Amide I, α-helix	1648–1666
Valine, Isoleucine	1124, 1160	Amide I, disordered	1660–1682
		Amide I, β-sheet	1662–1675

#### Conventional Raman Spectroscopy

Conventional Raman spectroscopy (also called spontaneous Raman spectroscopy) is the original Raman spectroscopy without any enhancement by other techniques. Hanlon et al. (2000) first used conventional Raman spectroscopy to analyze the distinction of brain tissues (including temporal cortex and white matter) between AD patients and normal subjects. The low-frequency shoulder on the 1670 cm^–1^ band in the spectrum of AD tissues was assigned to β-pleated sheet conformation of Aβ in senile plaques (SP), while the other two Raman transitions at 940 and 1150 cm^–1^ were not able to be assigned to any biomarkers. In order to get more persuasive results, Dong et al. (2003) compared the spectra of SP cores extracted from cortical gray matter from AD patients with synthetic Aβ fibrils, and the similar peaks in their spectra at around 1666 cm^–1^ demonstrated that Aβ is the dominant component in SP cores and is present as a β-sheet. A similar study conducted by Sudworth and Krasner (2004) verified Raman spectroscopy’s ability to identify Aβ originating from neuritic plaques. In order to investigate the spectra of early AD, Chen et al. (2009) injected Aβ_25__–__35_ into the hippocampus of rats. Compared with the spectra of normal rats, a frequency blue-shift in the amide I band representing the shift from α-helix to β-pleated sheet conformation of Aβ appeared in AD spectra. Conventional Raman spectroscopy is a reliable tool to recognize the Aβ fibrils through the typical spectra of corresponding secondary structures.

Compared with its capability to detect the protein backbone’s conformation, conventional Raman spectroscopy is more sensitive to the tertiary structure of the sidechains of proteins. As metal ions, e.g., Zn, Cu, and Fe, were associated with the aggregation of Aβ in other studies, the detailed structural information can be complemented with conventional Raman spectroscopy. A series of studies were conducted to examine the metal ions’ binding modes of synthetic Aβ_1__–__40_ and Aβ_1__–__16_ in solution and insoluble aggregates by conventional Raman spectroscopy, confirming metal-Aβ forms and their corresponding spectral features (Miura et al., 2000, 2001; Suzuki et al., 2001). Based on the spectra-structure correlations from previous studies, Dong employed conventional Raman to detect the metal-binding sites in SP cores extracted from AD patients and monitored the effectiveness of treatment with chelator ethylenediaminetetraacetate (EDTA) (Dong et al., 2003). The spectrum of SP cores showed increased intensity at 1604 and 1278 cm^–1^, which were, respectively, ascribed to Zn binding at the N_τ_ site of histidine side chains and Cu binding at the N_π_ site of histidine side chain as the disease progressed. For the spectrum of SP cores treated with EDTA, the decrease in relative intensity of the 1278 cm^–1^ band confirmed the EDTA’s function of removing the His-bound Cu (II), and the increased bandwidth at half-height of amide I band represented greater heterogeneity, suggesting that a chelator could be a possible opportunity for AD treatment. Aiming to eliminate the interference of the hydrophobic part of Aβ that promotes its aggregation, Miura et al. (2004) synthesized Aβ_3__–__9_ (Glu3-Phe4-Arg5-His6-Asp7-Ser8-Gly9), an N-terminal Aβ segment without the hydrophobic parts of the full-length Aβ. The Raman spectrum suggested that Cu(II) induced the aggregation of Aβ3-9 by binding Aβ_3__–__9_ at Glu3, His6, and Asp7. Therefore, the Cu(II) binding at the truncated N-terminus of Aβ could play a role in the mechanism of AD. Thus, conventional Raman spectroscopy’s ability to probe the coordination between residues and metal ions could lead to a more accurate and deeper understanding of the AD mechanism.

One disadvantage of conventional Raman spectroscopy is its weak signal due to weak Raman scattering, resulting in a much longer processing time than other spectroscopy techniques, limiting the use of Raman spectroscopy in AD diagnosis. In addition, conventional Raman spectroscopy cannot clearly distinguish parallel from the anti-parallel β-sheet (Martial et al., 2018). Subsequently, Raman-derived devices have been developed to address these limitations, including resonance Raman spectroscopy, surface-enhanced Raman spectroscopy (SERS), tip-enhanced Raman spectroscopy (TERS), and coherent Raman spectroscopy (CRS).

#### Resonance Raman Spectroscopy

In contrast with conventional Raman spectroscopy, resonance Raman spectroscopy selectively employs laser excitation frequency, which is close to the electronic transition of certain chemical bands of samples, contributing to a Raman signal enhancement of 10^6^–10^8^-fold (Oladepo et al., 2012). To advance the relative intensity of target bands, researchers choose corresponding laser wavelengths. For example, the amide bands can be typically resonantly enhanced by laser excitation with a deep UV wavelength around 195 nm (Lednev et al., 2005). Popova et al. (2010) employed the deep UV resonance Raman spectroscopy (DUVRR) to explore the aggregation of Aβ_1__–__40_ and the Aβ_34__–__42_ fragment. With the assist of hydrogen-deuterium exchange and an approach that estimates the dihedral angle psi (ψ) of protein and peptide amide groups with the UVRR amide III band frequency, the parallel β-sheet structure of fibrils from Aβ_1__–__40_ and the anti-parallel β-sheet structure of the fibrils from Aβ_34__–__42_ fragment were clearly distinguished. The aggregation mechanism of the Aβ_25__–__40_ fragment, the hydrophobic portion of Aβ_1__–__40,_ has also been studied with DUVRR (Xiong et al., 2014; Xiong and JiJi, 2017). Additionally, the kinetics of fibril formation of tau were investigated by UVRR with an excitation wavelength of 220 nm (Ramachandran et al., 2014). Another excitation wavelength of 413.1 nm was employed to investigate the binding of heme and Aβ and the characteristics of heme-Aβ complexes (Ghosh et al., 2013). In the investigation of fast kinetics of aggregation and binding process of pathological proteins, resonance Raman spectroscopy could be a powerful instrument with high structural and temporal resolution.

#### Surface-Enhanced Raman Spectroscopy

**FIGURE 4:**
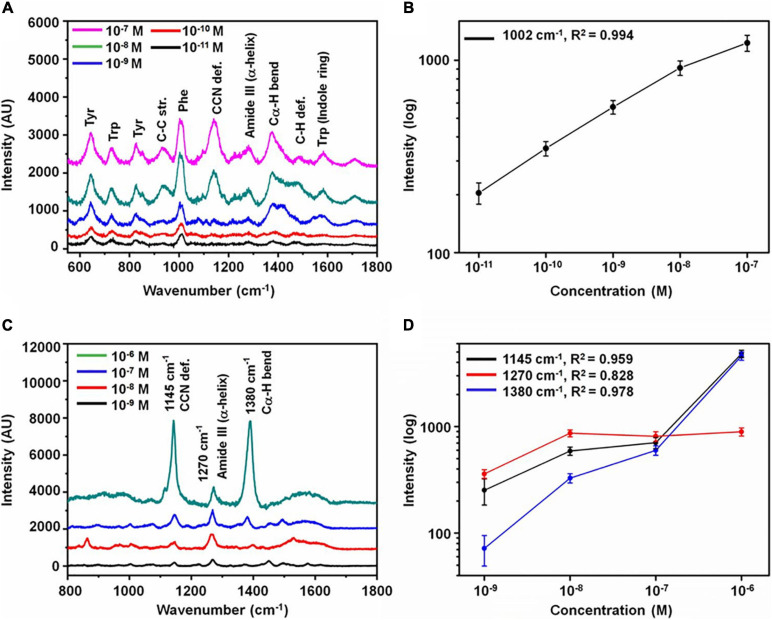
Analysis of Alzheimer’s disease biomarkers. **(A)** SERS spectra with various concentrations of tau protein and **(B)** variation of intensity at 1002 cm^–1^ as a function of concentration. **(C)** SERS spectra with various concentrations of Aβ and **(D)** variation of intensity at 1145, 1380, and 1270 cm^–1^ as a function of concentration (the error bars represent the standard deviation of 10 measurements at random spots). Reprinted with permission from Park et al. (2020). Copyright (2020) American Chemical Society.

Surface-enhanced Raman spectroscopy (SERS) can tremendously enhance the Raman signal by adsorption of molecules onto metal surfaces based on electromagnetic enhancement and chemical enhancement. In principle, the enhancement can be 10^10^–10^14^ orders of magnitude with the combination of electromagnetic enhancement and chemical enhancement (Pilot et al., 2019). The most prominent advantages of SERS are its high sensitivity and the fabrication of reproducible and robust substrates. Abundant studies have been performed to set up SERS platforms to detect AD biomarkers with high sensitivity. Chou et al. (2008) employed a nanofluidic device that transports the mixture of molecules and gold colloid particles (60 nm) to a nanochannel under the illumination of a SERS platform. This nanofluidic biosensor could detect Aβ_1__–__40_ at a concentration as low as 11.5 pM. Buividas et al. (2015) developed the laser fabricated ripple SERS substrates to provide a quantitative assessment for soluble Aβ_1__–__40_ oligomer with a concentration range of 10 nM–10 μM. Furthermore, Park et al. (2020) applied a carboxylic-acid-functionalized and graphitic nanolayer-coated three-dimensional SERS substrate (CGSS) on the quantitative detection of tau and Aβ (Figure 4). The concentration of tau and Aβ at the range of 10 pM–10 nM and 1 nM–100 nM, respectively, were accurately measured based on the intensity of the Phe band at 1002 cm^–1^ and the amide S-band at 1380 cm^–1^ in each spectrum. Zhou et al. (2020) designed a ratiometric label-free SERS platform to monitor the formation of Aβ_1__–__40_ fibrils from monomers with the generation of gold nanoparticles in brain tissues. This SERS platform achieved the reliable detection of Aβ_1__–__40_ monomers and Aβ_1__–__40_ fibrils above the concentration of 70 ± 4 and 3.0 ± 0.5 pM, respectively.

The high sensitivity of SERS is due to its ability to detect AD biomarkers at low concentrations and differentiate biomarkers or their shift at the molecular level. Yu et al. (2018) employed SERS to study the distinction of Aβ_1__–__40_ and Aβ_1__–__42_ and their assembly phases with a graphene-gold hybrid plasmonic platform. Coupled with principal component analysis (PCA), SERS could readily distinguish Aβ_1__–__40_ from Aβ_1__–__42_ and differentiate their assembly stages. In addition to the normal SERS, SERS active immunosensors were designed by combining substrates with antibodies of target biomarkers to improve the sensitivity, even achieving the detection of biomarkers at fM level concentration (Maurer et al., 2020; Yang et al., 2020). The high sensitivity of SERS may make it possible to be an effective method for early screening or diagnosis of AD. However, it is difficult to judge the obscure effects of exogenous reagents on biomarkers, and the impact of metal nanoparticles on biomarkers should be investigated to avoid the acquisition of false results. For instance, the contact between metal nanoparticles and proteins may lead to denaturation and dysfunction in certain proteins (Bruzas et al., 2018); specifically, the gold nanoparticles can inhibit the aggregation of Aβ (Mirsadeghi et al., 2015).

#### Tip-Enhanced Raman Spectroscopy

For tip-enhanced Raman spectroscopy (TERS), the combination of SERS and a scanning probe microscope, atomic force microscope (AFM), or scanning tunneling microscope (STM), can accomplish high sensitivity and nanoscale spatial resolution. Aiming at Aβ fibrils, several studies have been performed with TERS on the insulin fibril, a typical amyloid fibril model (Deckert-Gaudig et al., 2012, 2016; Kurouski et al., 2014). Bonhommeau et al. (2017) employed TERS to distinguish natural Aβ fibrils and two synthetic mutants [less toxic amyloid fibrils (L34T) and highly toxic oligomers (oG37C)]. With the examination of amide I and amide III bands, the parallel β-sheets structure of natural Aβ fibrils and L34T could be clearly distinguished from the anti-parallel β-sheets structure of oG37C. Furthermore, the correlation between nanoscale structure and toxicity could be explored by TERS (D’Andrea et al., 2018). In another study, TERS was conducted on Aβ oligomer-treated neural spines (Tabatabaei et al., 2017). While individual spines were imaged with AFM, the Aβ aggregations at the surface of each spine could also be detected under TERS measurement. The heterogeneous Aβ fibrils were indicated with the various TERS spectrum. Thus, TERS could be a powerful tool to promote the understanding of AD’s mechanism at the molecular level owing to its high sensitivity and excellent resolution.

#### Coherent Raman Spectroscopy

Unlike other Raman spectroscopy, coherent Raman spectroscopy (CRS) is based on the detection of coherent Raman scattering, which is formed under the illumination of two synchronized pulsed lasers called pump laser (ω_*p*_) and Stokes laser (ω_*S*_). When the frequency difference (ω_*p*_-ω_*S*_) between pump laser and Stokes laser corresponds with a molecular vibration mode, a resonant enhancement occurs for coherent anti-Stokes Raman scattering (CARS) at the frequency of “2ω_*p*_-ω_*S*_” and stimulated Raman scattering (SRS) at the frequency of “ω_*p*_” or “ω_*S*_.” The difference between CARS and SRS should be considered when researchers choose suitable tools for their specific aims. CARS has a natural limitation that the non-resonant optical effects cause background, but SRS does not, making SRS a quantitative and sensitive method. Coupling CRS with microscopy can ideally fix the long integration time of conventional Raman microscopy due to its high signal intensity and be applicable to image AD brain tissue.

### Raman Imaging in AD

Raman imaging is an excellent tool to generate images consisting of both spectral information and location information. According to the different imaging processes, Raman imaging can be divided into two imaging modes: Raman mapping and wide-field Raman imaging. For the Raman mapping, the Raman microspectrometer collects the whole Raman spectra of the region of interest (ROI) by focusing on a point or a line of this region every time. Then a false-color image can be constructed based on the analysis of Raman data. In wide-field Raman imaging, the spectral intensity at a specific wavenumber of the whole ROI is acquired simultaneously. According to the acquisition range of spectra, Raman imaging can be categorized into hyperspectral Raman imaging and direct Raman imaging. Raman imaging is still in the research phase for exploring the reliable and sensitive biomarkers for AD detection; thus, hyperspectral Raman imaging is more frequently used.

#### Raman Mapping for AD Detection in Brain Tissues

Raman mapping can recognize AD pathology by analyzing the whole spectrum of each point in the scan region. The analysis assists the exploration of specific features of the AD Raman spectrum and the combination of multiple weak features from the spectrum to form obvious differences to identify AD.

Michael et al. (2014) employed the Raman mapping consisting of confocal Raman microspectrometer and hierarchical cluster analysis on AD patients’ hippocampal tissues and lens tissues. For every sample with a size of 30 μm × 30 μm, the hierarchical 4 cluster analysis was applied on 4,096 spectra in the range of fingerprint (700–1800 cm^–1^) and high-frequency region (2700–3700 cm^–1^) with a dwell time of 50 ms/pixel. Each sample’s spectra were divided into four groups depending on their similarity. For the hippocampal samples, one of the significant distinctions between the 4 clusters is the intensity of the Raman signal at 1668 cm^–1^ assigned to the β-sheet configuration of proteins. In contrast, the spectra of lens tissues showed a minor intensity difference between the four groups and a much lower intensity of 1668 cm^–1^ suggesting the absence of Aβ in lens tissues. Based on the results of the previous study, the same team performed Raman mapping on twelve frontal cortex and hippocampus samples from three AD patients and one control donor (Michael et al., 2017). From the images, the neuritic plaques and neurofibrillary tangles can be identified with a spatial resolution of 0.47 μm. However, Lochocki questioned the authenticity of the imaging of neuritic plaques and neurofibrillary tangles due to lacking validation like immunohistochemical imaging and Thioflavin-S staining performed on the same samples (Lochocki et al., 2020). The team performed Raman imaging and Thioflavin-S staining on fixed brain tissue from three AD patients and two controls, and the Raman spectra at 1669 and 1445 cm^–1^, representing the β-sheet and lipids, respectively, were selected to form the intensity mapping. The Raman images showed no significant difference between the AD tissues and healthy control tissues, while Thioflavin-S stained images distinguished them clearly. Although it is hard to deny the potential of Raman imaging to detect AD pathology with the results of just one study, at least it reminds researchers to exclude the interference of autofluorescence in similar studies.

A study performed by Palombo et al. (2018) employed Raman imaging, micro-Fourier transform infrared (μFTIR) imaging, and immunofluorescence imaging to investigate the plaques of brain tissues from a *TASTPM* transgenic mouse model, which carries mutations on the *APP* and presenilin-1 gene. Principal Component Analysis (PCA) and Self Organizing Maps (SOM) algorithm were used in Raman imaging. The plaques and surrounding lipid rings were clearly visualized by Raman imaging and μFTIR imaging. The components of plaques were validated as Aβ by amylo-glo staining, and the lipid rings were correlated with the astrocyte processes by immunofluorescent staining of anti-Glial fibrillary acidic protein (GFAP) antibodies. Another gain of this research is the direct comparison between Raman imaging and μFTIR imaging in the AD field. Raman imaging has a better spatial resolution for imaging the cell bodies, while μFTIR imaging possesses a much higher imaging speed, which is more suitable for clinical utilization. To improve Raman imaging speed to accelerate the transition from the laboratory to the bedside, two options are available. The first one is the utilization of derived instruments to enhance the weak Raman signal. The second one is the adjustable spectrum of wide-field Raman imaging.

#### Wide-Field Raman Imaging for AD Detection in Brain Tissues

Wide-field Raman imaging, also called global Raman imaging, is a bit like taking photos. The images consist of one or multiple wavelengths of the spectra of molecules or structures, limited by the filtered illumination for the whole ROI. When it comes to a narrow part of the spectrum, less data record and analysis make it real-time imaging.

Previous studies reported that lipids accumulate in the region of the fibrillar plaque and play a role in the formation of fibrillar plaque. Kiskis et al. (2015) imaged the lipids around fibrillar plaques in AD patients’ brain tissues by coherent anti-stokes Raman scattering (CARS) microscopy with a high spatial resolution of 0.3 μm. The CARS images at 2840 cm^–1^, which represents the symmetric CH_2_ vibrations of lipids, showed the distribution of lipids in AD patients’ brain tissue sections to be consistent with the position of fibrillar Aβ plaques in the Thioflavin-S stained images. The lipid aggregates showed two morphological structures in CARS images, including multi-lamellar structures and coalescent structures. Furthermore, a CARS CH ratio image composed of the ratio of CARS signal at 2840 and 2870 cm^–1^ (representing asymmetric CH_2_ vibrations of lipids), imaged the fluidity and heterogeneity of lipids, which could be a biomarker for the detection and prognosis in AD.

**FIGURE 5:**
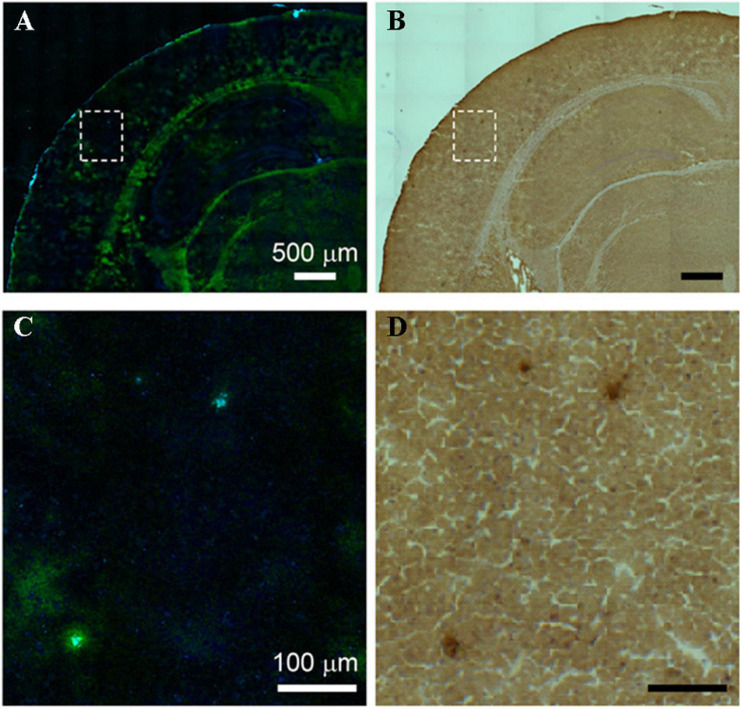
Comparison between SRS and antibody staining results on the same tissue section. **(A)** SRS image and **(B)** antibody staining image on the same AD brain tissue section, **(C,D)** zoom-in images of the dotted box regions in **(A,B)**. Reprinted with permission from Ji et al. (2018). Copyright (2018) The Authors, some rights reserved; exclusive licensee American Association for the Advancement of Science.

**FIGURE 6:**
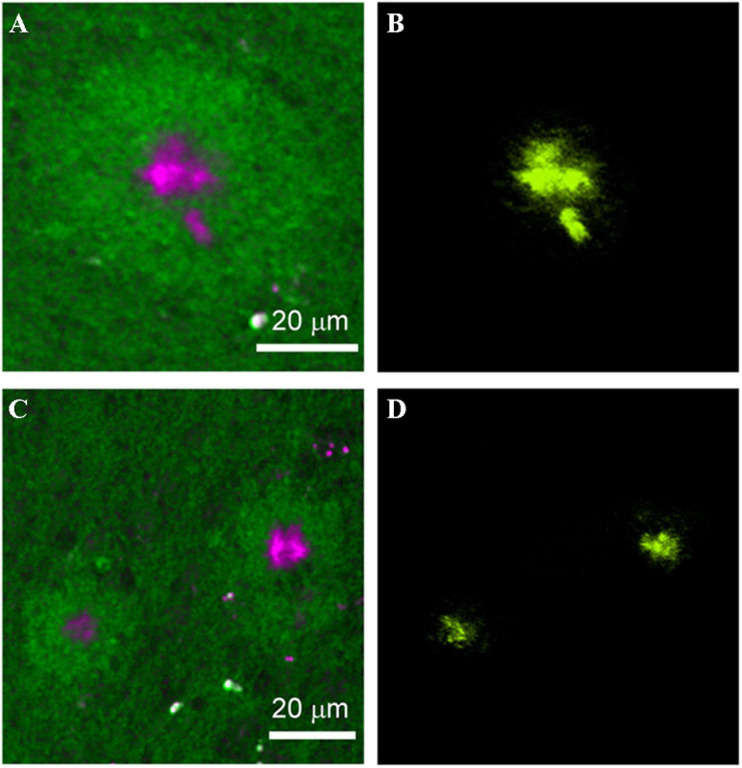
Comparison between the SRS and thioflavin S–labeled two-photon imaging results on the same tissue. **(A,C)** SRS images and **(B,D)** thioflavin S–labeled two-photon images on the same AD brain tissue. Reprinted with permission from Ji et al. (2018). Copyright (2018) The Authors, some rights reserved; exclusive licensee American Association for the Advancement of Science.

Stimulated Raman scattering (SRS) microscopy was employed to image amyloid plaques for frozen sections and fresh tissues from the brain of an AD transgenic mouse model with a high acquisition speed of 5 s/frame (Ji et al., 2018). The visualization of amyloid plaques in frozen sections and fresh tissues was verified by antibody staining and dye staining, respectively (Figures 5, 6). Further, the distinction between the images of frozen sections and fresh tissues suggested that tissue processing affected tissue’s structure, which should be avoided as much as possible. Compared with the staining image, Raman imaging shows superiority with its simple sample processing and the detection of more histological changes.

## Optical Coherence Tomography (OCT)

**FIGURE 7:**
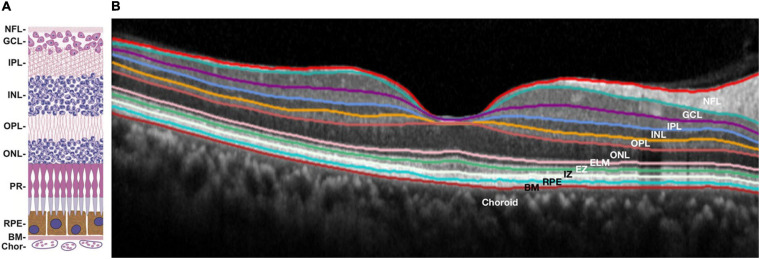
Structure of human retina: schematic diagram **(A)** and retinal optical coherence tomography image with segmented layers **(B)**. NFL, nerve fiber layer; GCL, ganglion cell layer; IPL, inner plexiform layer; INL, inner nuclear layer; OPL, outer plexiform layer; ONL, outer nuclear layer; ELM, external limiting membrane; PR, photoreceptors; EZ, ellipsoid zone; IZ, interdigitation zone; RPE, retinal pigment epithelium; BM, Bruch’s membrane; Chor, choroid. **(A)** reprinted with permission from Malek and Lad (2014). Copyright (2014) Springer Basel; **(B)** reprinted with permission from Ong et al. (2019). Copyright (2018) Springer-Verlag GmbH Germany, part of Springer Nature.

Optical coherence tomography (OCT) has been known for its extensive application in ophthalmology since the 1990s, when it was initially introduced to image the human retina (Huang et al., 1991). OCT is a rapid, non-invasive instrument that provides cross-sectional imaging of samples. Cross-sectional images are based on the light backscattered from samples, similar to ultrasonography. OCT is based on low coherence interferometry, which is the key process for transforming backscattered light into images. The initial OCT technique is Time-domain OCT (TD-OCT), and Spectral-domain OCT (SD-OCT) appeared on its heels. The latter made a significant improvement in resolution, sensitivity, scan speed, and signal-to-noise ratio, which contributed to clearer and more accurate images. With the rapid development of OCT, other functional extensions emerged successively, such as optical coherence microscopy (OCM) and optical coherence tomography angiography (OCT-A). Considering the advantages of OCT in intraocular imaging and the connection of AD with eyes, its application in AD screening and diagnosis is entirely conceivable (Figure 7).

### Conventional Optical Coherence Tomography in AD

The first appearance of OCT application in AD is when Parisi et al. (2001) employed OCT to measure the retinal nerve fiber layer (RNFL) of 17 AD patients and 14 age-matched controls and found there was a significant reduction for both overall and each quadrant of RNFL thickness in AD patients compared to controls. Many similar studies were conducted to verify the results. Not all of them showed the same result because of the variabilities in experiment subjects and OCT devices. For example, the statistically significant RNFL thinning from published articles vary at different locations, including the superior temporal sector (Cunha et al., 2017a), superior nasal sector (Kromer et al., 2014; Eraslan et al., 2015), superior quadrant (Kirbas et al., 2013; Liu et al., 2015; Kwon et al., 2017), superior and inferior quadrants (Lu et al., 2010; Kesler et al., 2011; Moschos et al., 2012), all quadrants except temporal (Iseri et al., 2006; Bambo et al., 2015), all quadrants except nasal (Gao et al., 2015), and all quadrants (Ascaso et al., 2014; Tao et al., 2019). Other parameters in the OCT results of the retina also indicated a meaningful distinction between AD subjects and controls, including macular volume (Ascaso et al., 2014; Gao et al., 2015; Salobrar-Garcia et al., 2015), Ganglion cell layer (GCL) thickness (Garcia-Martin et al., 2016), the inner plexiform layer (IPL) thickness (Garcia-Martin et al., 2016; Snyder et al., 2016), and ganglion cell-inner plexiform layer (GC-IPL) thickness (Cheung et al., 2015; Shao et al., 2018). Additionally, the choroid attracted tremendous attention as the vascular layer of the eye. Gharbiya et al. (2014) employed SD-OCT to measure the RNFL thickness and the choroidal thickness and found a significant reduction of choroidal thickness in AD patients compared to controls. After that, other relevant studies appeared successively to confirm this discovery (Bayhan et al., 2015; Bulut et al., 2016; Cunha et al., 2017b).

Lian et al. (2020) performed a comparative experiment between AD patients with abnormal and normal retinas. 96 AD patients were divided into 52 (54.17%) AD patients with an abnormal RNFL thickness and 44 (45.83%) AD patients with a normal RNFL thickness. Comprehensive AD symptom assessments were performed on all patients, including cognitive function, neuropsychiatric systems, and activities of daily living (ADL). Compared with the AD patients with normal RNFL thickness, those with abnormal RNFL thickness had significant impairment in cognitive function and ADL. In addition to the neurological symptoms, some physical brain changes can also be correlated with the RNFL thickness. Zhao et al. (2020) measured the RNFL thickness with OCT and hippocampal volume with MRI on AD patients, MCI patients, and normal subjects. Both the macular RNFL thickness and hippocampal volume show a significant decrease in the order of normal subjects, MCI patients, and AD patients, and the macular RNFL thickness showed a significant correlation with hippocampal volume. Another similar study performed by Carazo-Barrios et al. (2021) revealed that the mean RNFL thickness of left temporal quadrants was significantly correlated with the number of occipital white matter lesions. None of the aforementioned cross-sectional studies could dynamically describe these parameters such that further longitudinal studies are needed. Trebbastoni et al. (2016) organized a 12-month prospective study on 36 mild to moderate AD patients and 36 control subjects. In this study, all subjects experienced neuropsychological evaluation and SD-OCT measurement at the beginning and end. The results showed a significant reduction of the inferior and superior RNFL thickness in the AD group, and a correlation between reducing the inferior RNFL thickness and the patient’s cognitive decline. In another 12-month prospective study, the same team found a greater reduction of choroidal thickness in AD patients than in controls (Trebbastoni et al., 2017). Therefore, the usage of OCT for measuring eyes could be a potential tool for monitoring AD progression.

In an attempt to assess the early diagnosis potential of OCT, Armstrong et al. (2021) spent 5 years recruiting ten carriers with *PSEN1* gene mutation, which is the critical causative gene mutation of familiar early onset AD. Compared with the noncarrier group, the *PSEN1* mutation carriers showed a significant decrease in the retinal thickness of outer superior quadrant, outer nasal quadrant, outer temporal quadrant, inner superior quadrant, inner inferior quadrant, inner temporal quadrant, and some other individual retinal layers. Thus, it was shown that some OCT parameters could be reliable biomarkers for screening AD before its clinical onset.

In addition to the detection of abnormal structure of AD retina, OCT was used to image the specific pathological AD hallmarks. Kayabasi et al. (2014) employed the combination of OCT and fundus autofluorescence (FAF) on the eyes of 30 MCI patients. Dot-shaped bright depositions were seen in the nerve fiber, ganglion cell, and outer plexiform layers in OCT images and speculated as Aβ-rich depositions through curcumin staining. The study performed by Koronyo et al. (2017) also confirmed that the positions of Aβ depositions are above the retinal pigment epithelium (RPE) layer of AD patients using OCT. Besides, the approach of combination of OCT and FAF was utilized again on PET-proven AD patients to visualize neurofibrillary tangles (NFTs) by Kayabasi (2020). The ability of OCT to image Aβ depositions and NFTs, when combined with autofluorescence imaging, could provide a more specific and effective method to screen for AD, but more relevant studies are needed.

### Optical Coherence Microscopy

Optical coherence microscopy (OCM), as the name suggests, is OCT-based microscopy. It inherits the advantages of OCT, including high axial resolution and high signal-to-noise ratio, while also improving the lateral resolution by coupling with a high numerical aperture objective lens. OCM has been used for many neuroimaging applications (Srinivasan et al., 2012; Wang et al., 2016), but not for AD until 2012, when Aβ plaques were first imaged using OCM (Bolmont et al., 2012). In this study, Bolmont et al. utilized an extended focus OCM (xfOCM) to enable the distinction of the Aβ plaques from surrounding regions in fresh brain tissue of *APPPS1* transgenic AD mice with the axial resolution of 2 μm and the lateral resolution of 1.3 μm, both *in vivo* and *ex vivo*. The image contrast between Aβ plaques and surrounding brain tissue was due to their different light-scattering properties. Compared with the conventional OCM, in which a high lateral resolution and broad focal range cannot be attained simultaneously, the xfOCM achieves both with a Bessel beam. The imaging depth of xfOCM is around 600 μm, which is much higher than conventional OCM.

Based on the intrinsic birefringence of Aβ plaques, polarization-sensitive OCM (PS-OCM) was considered to improve contrast. Baumann et al. (2017) employed PS-OCM operating in the near-infrared wavelength region to successfully image neuritic Aβ plaques in AD patients’ brain samples. The laser sources with a broader spectrum and centered with a shorter wavelength was employed to realize higher axial resolution. Marchand et al. (2017) presented the visible spectrum optical coherence microscopy (visOCM) to visualize Aβ plaques. Using the broad spectral light source from the visible to the near-infrared wavelength range, the OCM attained an axial resolution of 0.69 μm and a lateral resolution of 0.4 μm. However, the imaging depth of visOCM was limited to around 110 μm beneath the sample surface due to the low penetration depth of visible light. In order to improve this limitation, Lichtenegger et al. (2017, 2018) applied an advanced clearing technique, called system-wide control of interaction time and kinetics of chemicals (SWITCH), on AD brain tissues to get a penetration depth of 200 μm. The main drawback is that it cannot be used *in vivo*. Thus, OCM may be a valuable three-dimensional imaging tool for AD brain tissue.

### Optical Coherence Tomography Angiography

Optical coherence tomography angiography (OCT-A) is a functional extension of OCT, that was developed to image microvasculature. Compared with conventional angiography (fluorescein angiography and indocyanine green angiography), OCT-A can produce angiographic images non-invasively based on natural contrast by detecting the blood motion within vessels. OCT-A has been used to investigate the cerebral vascular changes in several neurological diseases, like traumatic brain injury (TBI), stroke, and aging (Jia and Wang, 2011; Choi and Wang, 2017; Li et al., 2018). Considering the close connection between AD and cerebral vascular deficits (Klohs, 2019), the potential application of OCT-A in AD cerebral vascular changes should be considered.

Besides cerebral vascular deficits, the retinal vasculature changes in AD patients have been investigated by other tools (Cheung et al., 2014). To get more accurate microvascular images and a more reliable conclusion, OCT-A is needed. The first application of OCT-A for analyzing retinal vascular density in AD was published in 2017 (Bulut et al., 2018). Bulut et al. reported that the retinal vascular density in patients with Alzheimer’s type dementia was significantly lower than controls in the whole region, corresponding to a significantly enlarged foveal avascular zone (FAZ). With the rapid deepening of the relevant studies, other more precise and representative parameters were introduced. For instance, several OCT-A studies revealed lower densities of superficial capillary plexus (SCP) (Grewal et al., 2018; Jiang et al., 2018; Lahme et al., 2018; Yoon et al., 2019; Zhang et al., 2019; Wang et al., 2020) and deep capillary plexus (DCP) (Jiang et al., 2018; Zabel et al., 2019; Wu et al., 2020) in AD subjects. Several studies were conducted to explore the retinal microvasculature changes in preclinical AD and MCI to test OCTA’s capability of detecting AD in the early stage. Wu et al. (2020) reported a significant microvascular loss of DCP in patients with MCI. Another study showed a similar result but only for the macular region’s superior nasal quadrant (Jiang et al., 2018). In contrast, the other two studies found no significant difference in vascular density between MCI subjects and controls (Querques et al., 2019; Yoon et al., 2019). In preclinical AD patients, O’Bryhim et al. (2018) reported enlargement of FAZ, while a study with a larger sample size showed a reverse result with a significantly higher vessel density (van de Kreeke et al., 2020). Considering these mixed results, there is no clear indication of OCT-A’s role in the early diagnosis of AD. However, the ability of OCT-A for detecting the vascular shifts in eyes induced by AD is valuable.

## Autofluorescence Imaging (AFI)

Autofluorescence is the light naturally emitted by biological structures when they absorb light at certain wavelengths. It is an intrinsic characteristic based on the inherent properties of biomolecules. In many cases, autofluorescence is regarded as the impediment for imaging specific substances with fluorescent probes. For instance, many fluorescent probes have been developed to probe Aβ plaque in brain samples, including Congo Red (CR), Thioflavin T (ThT), curcumin, and some near infrared (NIR) probes (Fu and Cui, 2018). However, lipofuscins are puncta-like cellular waste accumulating in aged brain tissue and have intense autofluorescence emission (Porta, 2002; Terman and Brunk, 2004). Their existence also seriously interferes with other optical imaging techniques, such as Raman imaging, due to their broad excitation light (Lochocki et al., 2020). On the other hand, the autofluorescence properties of some AD-relevant biomarkers could serve as a powerful tool for AD diagnosis.

**FIGURE 8:**
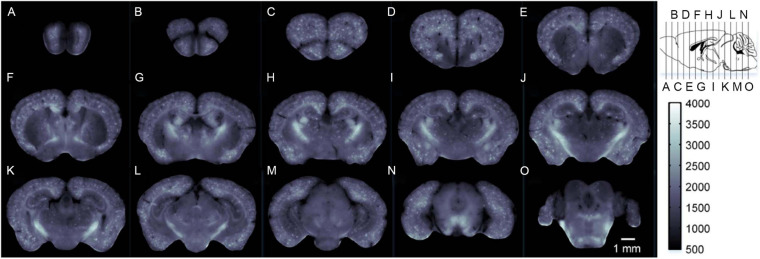
Label-free brain-wide map of senile plaque coronal distributions **(A–O)** obtained by cryo-MOST. Images were acquired from the olfactory bulb to the cerebellum with a 1 mm spacing. Specific locations are shown in the upper right inset. Reprinted with permission from Luo et al. (2017). Copyright (2017) Optical Society of America.

In the early 1980s, Dowson (1981) first reported that the senile plaques (SP) in the brain tissues from patients exhibited blue autofluorescence when irradiated with ultraviolet light. However, the compositions of SP with this property were not investigated. Another study narrowed the UV light-induced substance to amyloid plaques by comparing it with anti-Aβ immunostaining and suggested that non-Aβ-components associated with Aβ were more likely responsible for the autofluorescence rather than Aβ itself in amyloid plaques (Thal et al., 2002). This study further showed amyloid autofluorescence in cerebral amyloid angiopathy. In addition to human samples, the autofluorescence of SP has been found in fixed, cryoprotected, and 14 μm-thick brain sections of transgenic mice overexpressing the human amyloid precursor proteins (*PDAPP* mouse model) (Diez et al., 2000). With the aim of preserving the autofluorescence of SP, a multi-photon microscope was used on 400 μm-thick native brain slices and showed the autofluorescence SP in four transgenic AD mouse models (Kwan et al., 2009). Cryo-micro-optical sectioning tomography (cryo-MOST) was also employed to acquire the distribution of autofluorescence SP in the whole brain of transgenic AD mice (Figure 8; Luo et al., 2017). Gao et al. (2019) conducted a comprehensive study on senile plaques’ autofluorescence and showed its superiority over other fluorescent methods with its high signal-to-noise ratio, high sensitivity, and high reliability.

Other autofluorescence phenomena have been found to be associated with Alzheimer’s disease. A paper by Sun et al. (2001) described red autofluorescence of neurofibrillary tangles (NFTs) in brain tissue from AD patients after irradiating unstained paraffin-embedded sections with light around 550 nm. A different paper showed that NFTs exhibited blue autofluorescence with the excitation light at 700–800 nm by two-photon microscopy (Zipfel et al., 2003). Thus, the autofluorescence characteristics of NFTs need further investigation. Macular pigment (MP) is a mixture of the carotenoids lutein, zeaxanthin, and meso-zeaxanthin. Dual-wavelength autofluorescence was used to compare the optical density of MP between AD patients and normal subjects; significantly less MP was found in AD patients (Nolan et al., 2014). However, MP is just one of the pigments in the retina. The autofluorescence of the entire retina can be characterized by fluorescence lifetime imaging ophthalmoscopy (FLIO). Jentsch et al. (2015) conducted a study to investigate the autofluorescence features of AD patients’ retina with FLIO. The results indicated a significant correlation between FLIO parameters and cognitive status (e.g., MMSE score), as well as p-tau181 in the cerebrospinal fluid (CSF). The non-invasive and convenient FLIO could diagnose AD, motivating further studies with larger samples and good grouping.

Autofluorescence observation does not require exogenous agents or cause any damage to the sample tissue. The morphology and metabolic activity of samples are preserved almost perfectly and can even be used for other tests or procedures after autofluorescence observations. All of this makes autofluorescence imaging a non-invasive, rapid, reliable, and sensitive imaging tool. With the appearance of more related studies, autofluorescence will be a vital means for AD screening, diagnosis, and mechanistic investigation.

## Optical Harmonic Generation Imaging (OHGI)

Like autofluorescence, optical harmonic generation imaging is an optical imaging technique based on the intrinsic emission of samples. As the most general harmonic generation, second-harmonic generation (SHG) is the optical phenomenon in which two photons with the same frequency react with nonlinear materials to produce a new photon with doubled frequency, halved wavelength, and doubled energy. This was first found in a quartz sample in 1961 (Franken et al., 1961). The generation of the SHG signal depends on the second-order non-linear susceptibility χ(2) of the sample. Specifically, it is only found in the non-centrosymmetric molecules (e.g., collagen and microtubule) (Lim, 2019). In contrast, third-harmonic generation (THG) relies on the third-order non-linear susceptibility χ(3) of samples. It arises in all materials because all materials have non-zero third-order susceptibility. However, at the same irradiation condition, the THG signal is weaker than the SHG signal, as determined by the value of χ(2) and χ(3) (Sun, 2005). Optical harmonic generation is a polarization-induced process rather than absorption-induced, which means that no additional energy is accumulated in the sample, resulting in almost no negative effects (e.g., photobleaching and phototoxicity) on the sample, making it a completely non-invasive imaging technique (Campagnola, 2011).

Second-harmonic generation is highly emitted from collagen and microtubules. As such, it is well applied in functional imaging of brain tissue for recognizing pathological lesions, especially for AD. Lee et al. (2015) imaged the collagen in hippocampal cornus ammonis-1 (CA-1) and dentate gyrus (DG) regions of unstained brain tissues from *5× FAD* mice and wild-type mice by detecting the SHG signal. The SHG microscopy results showed higher amounts of collagen in *5× FAD* mice than wild-type mice, prominently in the DG region. Additionally, as a microtubule-associated protein in AD pathology, tau is expected to be detectable by SHG, which was validated by a cell-culture study (Stoothoff et al., 2008). The increased SHG signal was detected in the tau-transfected neurons with an 800 nm excitation laser. Kwan et al. (2009) first used SHG microscopy to image the acute slices of brain tissues of multiple AD mouse models, including *APP Swedish*, *APPSwe/PS1*, *APPSwe/TauJNPL3*, and *APPSwe/PS1/Tau* mice. In this study, the dendritic microtubules in the CA-1 and senile plaques of the hippocampus were imaged by SHG. The measured length and number density of polarized microtubule arrays showed no statistically significant differences between AD and wild-type mice in regions near senile plaques and far from senile plaques, suggesting no change in the polarity and the morphology of dendritic microtubules in AD mouse models. However, another interesting and meaningful result was that the Aβ plaques emitted SHG signals, validated by the Thioflavin-S staining. THG has been further utilized in this research area as a complementary tool for SHG. Chakraborty et al. (2020b) employed THG microscopy to image the brain tissue from a *3 × Tg* AD mouse. The visualization of Aβ plaques by THG was validated by comparison with antibody staining images. Compared with the staining image, THG could visualize more detailed brain structures, such as neuronal soma and axon fiber bundles. By taking advantage of the emission of SHG from Aβ plaques and NFTs, and THG from Aβ plaques, the same research team used a combination of SHG and THG for differential visualization of Aβ plaques and NFTs (Chakraborty et al., 2020a). In this additive-color multi-harmonic generation imaging, the THG-green and SHG-red pseudo-colors were used to generate the images that visualize the yellow Aβ plaques, orange-red NFTs, and green axons and dendrites.

## Other Imaging Techniques

### Photoacoustic Imaging

**FIGURE 9:**
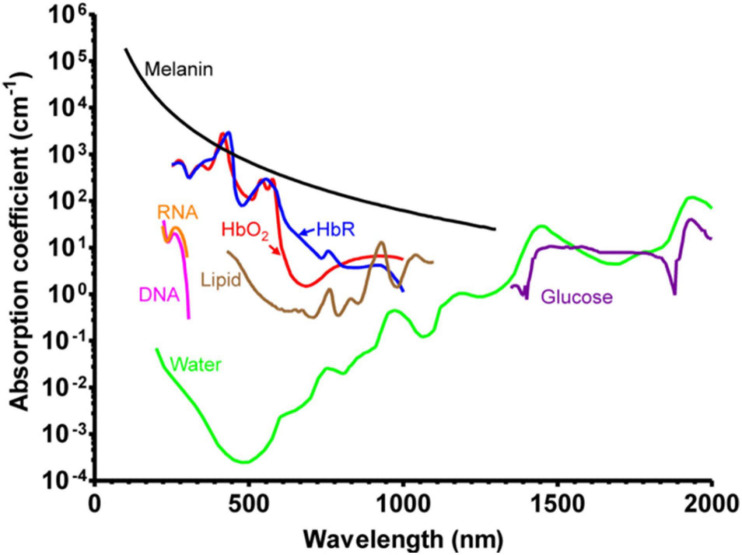
Absorption spectra of major endogenous chromophores in biological tissue. HbO_2_, red line (150 g/L in blood); HbR, blue line (150 g/L in blood); Lipid, brown line (20% by volume in tissue); Water, green line (80% by volume in tissue); DNA, magenta line (1 g/L in cell nuclei); RNA, orange line (1 g/L in cell nuclei); Melanin, black line (14.3 g/L in medium human skin); Glucose, purple line (720 mg/L in blood). Reprinted with permission from Yao and Wang (2013). Copyright (2012) WILEY-VCH Verlag GmbH & Co. KGaA, Weinheim.

Photoacoustic imaging, also called optoacoustic imaging, is recognized as a label-free tool for the visualization of hemodynamics. This technique is based on the photoacoustic effect, first reported by Alexander Graham Bell in 1880 (Bell, 1880). When the sample is illuminated with pulsed or modulated light, part of the absorbed light energy is converted into heat energy, causing the nearby tissues to undergo thermoelastic expansion, thereby forming ultrasonic emission. For the imaging of biological tissues, the acoustic signal is detected by the ultrasound transducer. The absorption spectra of major endogenous chromophores in biological tissues are described in Yao and Wang (2013) review (Figure 9). In the wavelength range of 250 to 830 nm, hemoglobin (Hb) has much higher absorption than other chromophores, except melanin. Oxy-hemoglobin (HbO_2_) and deoxy-hemoglobin (HbR) possess significantly different absorption coefficients around 700 nm wavelength, allowing the imaging of hemodynamics, including oxygen saturation of hemoglobin (sO_2_), blood flow, and oxygen metabolism, especially for cerebral vessels. Thanks to the thin skulls of rodents, photoacoustic imaging has been used to image and measure cerebrovascular structure, cerebral blood flow (CBF), sO_2_, oxygen extraction fraction (OEF), and the cerebral metabolic rate of oxygen (CMRO_2_) in living mice, including awake mice and anesthetized mice (Cao et al., 2017).

Hu et al. (2009) utilized optical-resolution photoacoustic microscopy (OR-PAM) as an imaging tool to visualize amyloid plaques in a living *APP/PS1* mouse. The ability of OR-PAM to image both amyloid-specific Congo red dye (exogenous chromophores) and hemoglobin (endogenous chromophores) provides the visualization of the amyloid plaques and surrounding blood vessels. In another study, Ni et al. (2018) used photoacoustic tomography as a label-free tool for imaging cerebral hemodynamics in arcAβ mice. The authors employed PAT to investigate cerebral oxygenation and MRI to assess the CBF in arcAβ and wild-type mice of 6- and 24-months of age. The 24-month old arcAβ mice had significantly lower CMRO_2_ than age-matched wild-type mice and 6-months old arcAβ mice. The OEF of all mice was similar. At the early phase of AD, CMRO_2_ was maintained in the normal range. At the advanced stage of the disease, CMRO_2_ decreased significantly without compensation from OEF. Therefore, CMRO_2_ could be a vital parameter for monitoring the progress of AD. CMRO_2_ can be measured just by the PAI system without the assistance of MRI (Cao et al., 2017), which means that PAI could image the hemodynamics and measure the oxygen metabolism of AD brains to monitor disease progression.

### Quantitative Phase Imaging

As a quantitative label-free optical imaging tool, quantitative phase imaging (QPI) has developed rapidly for high resolution and sensitivity in recent years. The ability of QPI to detect the intrinsic contrast of samples is based on the phase shift that occurs when the light travels through the transparent samples. The phase shift is closely connected with the inherent characteristics of the samples, particularly thickness and refractive index. Thus, detailed structural information of samples can be provided in the quantitative phase images, which consist of the measured local phase shift in each pixel, in the form of scattering parameters, such as scattering mean free path (ls) and anisotropy factor (g), obtained through the scattering-phase theorem (Wang et al., 2011). Multiple quantitative phase microscopes have been generated and advanced for cell morphology and pathology in biomedicine, including Digital Holographic Microscopy (DHM), Diffraction Phase Microscopy (DPM), Spatial Light Interference Microscopy (SLIM), etc. (Mir et al., 2012). In neuroscience, QPI has been used to image the whole mice brain slices by utilizing a motorized stage (Min et al., 2016). At the cell level (micro/nano-scale), QPI enables the differentiation of membrane, axon, and organelles to reconstruct the morphology of whole neurons. At the anatomical level (macro-scale), the detailed structures in the isocortex, olfactory bulb, hippocampus, cerebellum, choroid plexus, and corpus callosum are delineated based on the scattering properties of sample compositions. Therefore, QPI could be potentially applied for recognizing the neuropathology of neurodegenerative diseases, especially for AD.

Lee et al. (2016) utilized wide-field QPI on brain slices to investigate the pathological change of AD mice. Wide-field QPI was performed on AD-affected mice and their wild-type littermates by coupling DPM with a motorized XY-scanning stage, and the scattering coefficients (μs) and g maps were retrieved by a modified scattering-phase theorem. For both AD mice and wild-type mice, the gray matter, white matter, and hippocampi could be clearly distinguished in the μs maps and g maps according to the matched H&E-stained images. The key finding was that the higher level of μs value and g value throughout the gray matter and hippocampi regions in AD mice revealed higher inhomogeneity and increased size of scattering particles, which was not seen in staining methods and could possibly be structural biomarkers for AD diagnosis.

## Discussion

The aim of this review is to highlight the potential of the emerging label-free optical imaging techniques in clinical screening, diagnosis, and treatment of AD by presenting their imaging principles and emerging applications in AD. An essential characteristic of label-free imaging is preserving the original properties of samples without any exogenous agents, which means they could provide the most accurate and reliable results with less external disturbance in AD research, especially for AD mechanism and therapeutics studies. All the optical imaging techniques reviewed here have characteristics that contribute unique roles in various Alzheimer’s studies (Table 3). In addition to their imaging capabilities, the scanning cost, which is a pragmatic feature, should be considered. As clinical neuroimaging examination using an MRI or PET-CT could cost up to several thousand dollars, bringing an enormous economic burden on early AD diagnosis and making screening for high-risk patient populations infeasible. Conversely, the low cost of label-free, non-invasive optical imaging supports implementation for large-scale screening of high-risk populations and facilitates early detection and diagnosis of AD. Early detection and diagnosis provide patients an opportunity to participate in clinical trials that could target the disease in its early stages before irreversible brain damage or mental decline has occurred, construct a medical care team, plan advanced health directives, make financial and legal arrangements before significant cognitive decline. It also benefits physicians better manage a patient’s comorbidities and avoids prescribing medications that may worsen cognitive function.

**TABLE 3:** Comparison of salient features of the label-free optical imaging modalities for AD.

Imaging Techniques	Spatial Resolution	Sample Preparation	Biomarkers in AD	Advantages
FT-IR spectroscopic imaging	0.54–12 μm	***Ex vivo*** Flash-freezing; Fixation and freezing	Aβ plaques; Lipids; Creatine	Rapid; High specificity; High resolution.
Raman spectroscopic imaging	0.3–0.5 μm, lateral; 1–2 μm, axial	***Ex vivo*** Fresh tissues; Fixation and flash-freezing	Aβ plaques; Lipids	Real-time; High specificity; High resolution; Three-dimensional imaging; Minimal sample preparation.
OCT	>5 μm	***In vivo***	Ocular structures	Rapid; Non-invasive; Minimal sample preparation; Cost-effective.
OCM	0.4–2 μm, lateral; 0.88–5.6 μm, axial	***Ex vivo*** Flash-freezing; Fixation; Fixation and optical clearance	Aβ plaques	High resolution; Three-dimensional imaging; Minimal sample preparation.
OCT-A	>5 μm	***In vivo***	Retinal vascular density	Rapid; Non-invasive; Minimal sample preparation.
Autofluorescence imaging	−	***Ex vivo*** Fresh tissues; Fixation	Aβ plaques; NFTs; Macular pigment	Rapid; Simple; Low-cost; High specificity.
Optical harmonic generation imaging	0.31–0.57 μm^3^, 3D	***Ex vivo*** Fresh tissues; Fixation; ***In vitro***	Aβ plaques; NFTs; Collagen	Rapid; High specificity; High resolution; Three-dimensional imaging; Low-cost.
Photoacoustic imaging	5–150 μm	***In vivo***	Blood oxygenation	High sensitivity; Deep imaging depth.
Quantitative phase imaging	>0.8 μm	***Ex vivo*** Fixation and dehydration	Refractive index	Rapid; High resolution; Quantifiable; High sensitivity.

Meanwhile, the less cost, smaller size, and less technical support required label-free optical imaging devices can also be operated in diagnostic eye centers or ophthalmology clinics outside the hospitals and thus would be more conveniently accessible by the patients.

Compared with the successful application of label-free optical imaging techniques in AD research, these modalities still face certain hurdles before they can be considered completely non-invasive and are applicable to the screening and diagnosis of AD. For directly imaging AD-relevant fibrils in the brains of living patients, the most troublesome issue is the skull, which prevents the laser from reaching the brain. In animal-model research experiments, there are several methods to address the skull issue, such as craniotomy, cranial windows, skull-thinning techniques, and artificial skull implantation. Unfortunately, these invasive methods are unacceptable and actually do more harm than good for patients. Hanlon et al. (2000) proposed a nasal passageway for optical fiber probes to image the brain. Theoretically, the light from the optical fiber probe positioned at the olfactory epithelium can pass through the perforated cribriform plate and illuminate the olfactory bulb and the base of the frontal lobe. However, no relevant update has been reported. The utilization of a suitable IR light source may get around the skull barrier. Four skull transparency windows have been investigated for the transcranial light applications in rats, including NIR-I (700–1000 nm), NIR-II (1000–1350 nm), short-wave IR (SWIR) (1550–1870 nm), and SWIR II (2100–2300 nm) (Golovynskyi et al., 2018). Considering the high absorption of water in the SWIR region, NIR-I and NIR-II are likely better choices. Therefore, imaging requires that both excitation light and collected light coincide in the NIR region. As far as we know, no AD-relevant autofluorescence has been found in the NIR region. As for Raman techniques, the near-IR light sources have been utilized in various research areas, including brain imaging (Mizuno et al., 1992; Krafft et al., 2005), without the interference of autofluorescence compared with visible light. However, certain technical issues (e.g., weak Raman signal) in this process still need to be resolved with the development of Raman techniques before the realization of imaging the brain through the skull. In contrast, IR spectroscopic imaging could be possible for this purpose as it has a much higher signal than Raman.

Currently, it seems like the most feasible option for applying label-free optical imaging for AD in the clinic is indirect imaging, especially ocular imaging. As an extension of the brain, the retina has shown AD-related structure shift easily detected by OCT. The main thing that hinders its clinical utility is the lack of specificity for AD. Both glaucoma and other neurodegenerative disorders can lead to a similar variation on retinas, which are hard to differentiate (Moreno-Ramos et al., 2013; Eraslan et al., 2015). More clinical trials are warranted on AD patients, patients with other similar diseases, and normal subjects for building a database of the ocular structure changes in AD to develop AD diagnosis standards. Since the retina shares similar AD pathologies with the brain, even earlier detectable than in the brain (Koronyo-Hamaoui et al., 2011), the possible application of other label-free imaging techniques on AD retina investigation could be worth looking into. Stiebing et al. (2020) performed Raman imaging on unstained retinas of AD *APPswe/PS 1dE9* mice and controls to investigate the possibility of Raman imaging in AD screening through eyes. The cross-sectional Raman maps of retinas clearly identified the retinal layers and corresponded well with H&E staining images. The model established using chemometric analysis of *en face* retinas’ Raman spectra identified AD retinas with a sensitivity of 86.2%. However, no amyloid β deposition-attributed signal was found in AD retinas’ Raman images. This could have possibly been due to the absence of amyloid β deposition in retinas of this AD mouse model, seen from the western blot results of retinas. The combination of resonance Raman spectroscopy and fundus camera has also been applied for monitoring the macular pigment density in humans (Tanito et al., 2012), suggesting that Raman imaging could be a non-invasive and effective tool for AD screening through eyes.

Label-free optical imaging techniques are currently used in a guiding role for clinical operations of a variety of other conditions. Owing to their high resolution, real-time imaging, and reagent-free processing, these label-free imaging modalities have been applied in dermatology (Kong et al., 2013), ophthalmology (Hattenbach et al., 2016), breast cancer (Barman et al., 2013), and brain tumors (Hollon et al., 2016). Yuan et al. (2020) reported a microneedle (580 μm in outer diameter) combining OCT with laser ablation. In this study, OCT achieved high resolution (1.7 μm axial and 5.7 μm transverse), high imaging depth (1.23 mm), and high imaging speed (20 frames per second) in the process of brain tumor ablation. Although there is no surgical indication for AD patients, certain memory disorder therapeutics under investigation are directly delivered to the brain, such as gene therapy (Loera-Valencia et al., 2018; Raikwar et al., 2018) and deep brain stimulation (Xu and Ponce, 2017; Yu et al., 2019). Releasing at the right place and avoiding brain damage during the surgical operation are the key points of these treatment methods. As early as 2005, OCT was proved to image gray matter, white matter, and cerebral vessel clearly, indicating its potential as a practical guidance system of deep brain stimulation (Jafri et al., 2005). DePaoli et al. (2018) created a coherent anti-Stokes Raman scattering (CARS) probe with an outer diameter of 250 μm, which could be installed for guidance. This probe was tested in intact non-human primate brains and imaged the gray matter and white matter with high spatial resolution.

## Conclusion

In summary, this review provides a comprehensive overview of different classes of label-free optical imaging techniques that offer high resolution, reagent-free, cost-effective, and rapid imaging in revealing metabolic, morphologic, chemical, and structural attributes at the cellular level in live animal models or human specimens of Alzheimer’s disease. The review discusses the strengths and weaknesses of each of the label-free imaging techniques for early detection and diagnosis of Alzheimer’s disease that will benefit the patients and their families as well as the physicians who provide the care and manage the comorbidities. OCT has been approved for use in clinics for other disease applications. However, the modality has limited spatial resolution and molecular characterizations relevant to AD and lacks a standardized database to verify its efficacy. This prompts the question that a pragmatic label-free imaging system for AD diagnosis or treatment guidance may require the combination of two or more imaging modalities to be effective. The utilization of these label-free imaging tools for AD screening, diagnosis, and disease management is under rapid development. Further studies for data accumulation and device optimization (e.g., miniaturization) are needed for the translation into clinics.

## Author Contributions

SW designed the review. KL and JL co-wrote and edited the content. HZ, XL, RR, and SW contributed to the content and edited the draft. All authors read and approved the final manuscript.

## Conflict of Interest

The authors declare that the research was conducted in the absence of any commercial or financial relationships that could be construed as a potential conflict of interest.
